# When I say …. Respectful curiosity

**DOI:** 10.1111/medu.15552

**Published:** 2024-10-14

**Authors:** Amaya Ellawala, Enam Haque

**Affiliations:** ^1^ Hull York Medical School University of York Heslington UK; ^2^ Division of Medical Education University of Manchester Manchester UK

## Abstract

In the latest, “When I Say...” @AmayaEllawala and @enamhaque31 wrestle with the term ‘respectful curiosity’ to clarify its implications for health professions education and practice.

‘Replace judgement with curiosity’— Lynn Nottage, 2022

## 1 HISTORICAL CONTEXT AND IMPLICATIONS

Discrimination and bias are profound issues facing society. Within health professions education and health care, we constantly grapple with these issues as we strive to create equitable, diverse and inclusive spaces where the sense of belonging is mutual for students, teachers and patients.

While a heightened awareness about these issues is crucial in tackling issues of discrimination, it has inadvertently created a wariness in how people interact with each other. Where, in the past, it would not have been out of the norm to ask a new acquaintance ‘where are you from?’, questions such as this are now considered to have discriminatory undertones. As such, there is constant worry about the questions we ask and the words we speak, a fear that they may be perceived as offensive. This creates barriers between individuals as they shy away from questions that may seem intrusive and as a result, fail to learn about the people who surround them.

This can have vast implications in the health professions. A lack of awareness about individual students, patients and colleagues can negatively impact educational experience, clinical care and workplace relationships.

## 2 WHAT IS RESPECTFUL CURIOSITY?

Respectful curiosity was first described by the author Caroline Kettlewell,^1^ within a context of responding to self‐injury. The term has since permeated into wider spheres including health care and health professions education. Whitlock and Purington describe respectful curiosity as ‘a state of awareness characterized by a genuine curiosity and willingness to know and understand in combination with attention to assuring that one's curiosity is satisfied in a kind and respectful way’.^2^(p.1) We agree with this definition, particularly with the notion of ensuring that curiosity arises from a place of *genuine* interest and the necessity for ascertaining psychological safety. We see this as a skill that requires a cultivated lifelong learning approach, acknowledging that there is no absolute that can be attained, similar to cultural competency. Though a seemingly simple concept, we recognise that respectful curiosity can be complexly nuanced and have profound implications, both of which we will explore in the following sections.

## 3 BEING RESPECTFULLY CURIOUS

So how can we be respectfully curious in the health care and health professions education settings? We will illustrate this concept using the health care environment. In a patient consultation, we first need to look at ourselves and the bias and assumptions that we hold, following which, we must challenge our assumptions and use the patient encounter as an opportunity to actively listen to a patient and find out their agenda.^3^ The aim of this patient centred approach is to see a patient as an individual, develop empathy and build rapport with them, to enhance their care.^3^ Respectful curiosity can challenge injustices for minoritised groups, where normally their experiences of pain or other symptoms may be ignored, due to prejudice and assumptions by clinicians.^4^

A caveat to the curious approach is that it can become intrusive, with individuals exploring issues for their own gain, rather than for the benefit of others. When occurring within the context of patient care, this may breach confidentiality and encroach upon a patient's personal information.^4^ There is a fine line between respectful curiosity and being intrusive, and individuals need to ensure they remain on the correct side of the line. A key method of doing so is to keep checking themselves, to ensure the questions they ask are authentic and helpful and to continually gauge how recipients respond to the questioning. We can apply this approach to any setting if there is psychological safety in the learning environment. This is when someone feels free to highlight any mistakes or issues caused by individuals or the organisation, without the risk of repercussions.^5^

The following is an example of respectfully curious language in the clinical setting:

‘It sometimes helps with finding out the cause of your symptoms, to explore if you work and if so, what this involves. I hope you do not mind me asking this?”

(Wait for consent from patient)

‘Thanks. Are you working, and if so, what type of work do you do?’

The following language could be used in the higher education setting:

‘I am interested in learning more about my students, as this helps me create a better learning experience for them. I would be interested in knowing where you may be from. This would help me better understand your learning needs. I hope you do not mind, but out of this interest, could I ask where you are from?’

It is important to remember, however, that in order to build trust and mutual understanding, respectful curiosity should not be restricted to questioning alone but must include sharing something of oneself. As such, in the latter example, the teacher might add ‘to develop a strong educational relationship, it is important that we *both* get to know each other, so I hope you don't mind if I share something about myself?’

## 4 IMPLICATIONS OF ENGAGING IN RESPECTFUL CURIOSITY

So why is respectful curiosity so important? It is a key component of cultural humility, which is important in consultations and interactions with people from any background different to one's own. Foronda et al.,^6^ found that cultural humility requires an attitudinal shift in a person, ensuring that they are open to interacting with someone from a different culture, as well as being self‐aware of their biases and assumptions. It also encompasses removing ego and self‐reflection on any interaction with others. Cultural humility and respectful curiosity promote inclusive environments for learning, teaching and health care practice. This aligns with the Medical Schools Council (MSC) guidance on active inclusion and challenging exclusions,^7^ and with the General Medical Council (GMC) professional standards.^8^ It is evident therefore that respectful curiosity is vital in developing a shared understanding about each other and, as a result, in bringing communities together.

## AUTHOR CONTRIBUTIONS

**Amaya Ellawala:** Conceptualization; writing—original draft. **Enam Haque:** Conceptualization; writing—original draft.

## CONFLICT OF INTEREST STATEMENT

The authors have no conflicts of interest to disclose.

## Data Availability

Data sharing is not applicable to this article as no new data were created or analyzed in this study.

## References

[1] Kettlewell C . Skin game: a memoir. St Martin's Press; 2000.

[2] Whitlock J , Purington M . Respectful curiosity. In: The practical matters series, Cornell research program on self‐injury and recovery. Cornell University; 2013.

[3] Bansal A . Turning cross‐cultural medical education on its head: learning about ourselves and developing respectful curiosity. Fam Med Community Health. 2016;4(2):41‐44. doi:10.15212/FMCH.2016.0109

[4] Cheung K . Using curiosity to render the invisible, visible. Theor Med Bioeth. 2024;20:1‐9. 10.1007/s11017-024-09665-3 38767830

[5] Torralba KD , Jose D , Byrne J . Psychological safety, the hidden curriculum, and ambiguity in medicine. Clin Rheumatol. 2020;39(3):667‐671. doi:10.1007/s10067-019-04889-4 31902031

[6] Foronda C , Baptiste DL , Reinholdt MM , Ousman K . Cultural humility: a concept analysis. J Transcult Nurs. 2016;27(3):210‐217. doi:10.1177/1043659615592677 26122618

[7] Medical Schools Council . Active inclusion. Challenging exclusions in medical education. 2021.

[8] General Medical Council . Good Medical Practice. 2024.

